# Enhancing Communication Competence in Healthcare: The Impact of a One-Day Adventure-Based Experiential Learning Program

**DOI:** 10.7759/cureus.87554

**Published:** 2025-07-08

**Authors:** Yogesh Manickam Dominic Savio, Thirumoorthi Natarajan, Sujatha Lakshminarayanan, Madhumita Jeyalalitha Rajendran, Hariharan Surathkumaar, Ashni Bhandari

**Affiliations:** 1 Community Medicine, Sree Balaji Medical College & Hospital, Chennai, IND; 2 Department of Surgical Gastroenterology, Cancer Institute (Women's India Association), Chennai, IND; 3 Biostatistics and Epidemiology, Sree Balaji Medical College & Hospital, Chennai, IND

**Keywords:** adventure-based experiential learning, clinical communication skills, communication competence, experiential learning, healthcare professionals, interprofessional education, medical training, patient safety, simulation-based training, teamwork

## Abstract

Introduction

Effective communication is essential in healthcare, directly influencing patient safety, clinical outcomes, and team collaboration. Yet, traditional training approaches often fall short in delivering the practical skills required for high-pressure clinical environments. This study evaluated the effectiveness of a one-day adventure-based experiential learning (AEL) program in enhancing communication competence among healthcare professionals.

Methods

A quasi-experimental pre-test/post-test study was conducted at a tertiary care hospital with 500 healthcare professionals. The study was conducted at a private tertiary care teaching hospital in Chennai, India, over three months from May to July 2024.* *Participants were randomly assigned to either an experimental group (n=250, 50%) that completed a one-day AEL program or a control group (n=250, 50.0%) that received standard communication training. The sample included physicians (n=158, 31.6%), nurses (n=202, 40.4%), and allied health professionals (n=140, 28%). Gender distribution included 225 male (45%) and 275 female (55%) participants. Ages ranged from 23-30 years (n=175, 35%), 31-40 (n=195, 39.0%), 41-50 (n=95, 19%), and over 51 (n=35, 7%). Communication competence was measured using the Communicative Adaptability Scale (CAS), which evaluates six communication domains. These dimensions of interpersonal communication competence include social experience (comfort in varied social interactions), social confirmation (ability to validate and support others), social composure (management of anxiety and confidence in interactions), appropriate disclosure (judgment in sharing personal information), articulation (clarity and fluency in verbal expression), and wit (use of humor to facilitate communication). Each domain is measured using a 5-point Likert scale, with higher scores indicating greater communicative competence. Paired t-tests and analysis of covariance (ANCOVA) were used to assess outcomes, and effect sizes were calculated using Cohen’s d.

Results

Post-intervention, the experimental group showed statistically significant improvements in five of the six CAS dimensions. The most substantial gains were observed in social composure (d=0.85, large), social experience (d=0.82, large), and social confirmation (d=0.78, large), indicating strong practical significance (p<0.001 for all). The overall CAS score improved by 11.8% in the experimental group (from 3.47 to 3.88), compared to only 0.9% in the control group (from 3.45 to 3.48). ANCOVA analysis confirmed the effectiveness of the intervention, adjusting for baseline equivalence.

Conclusion

AEL is an effective strategy for enhancing communication competence in healthcare professionals. The program’s immersive and collaborative format improved the confidence, clarity, and adaptability of the participants. These findings support the integration of AEL into both medical education and professional development to strengthen interprofessional communication and improve patient care.

## Introduction

Effective communication is a foundational pillar of high-quality healthcare, influencing clinical outcomes, patient satisfaction, and safety across all levels of care delivery [1]. From diagnostic accuracy to treatment adherence and interdisciplinary collaboration, the ability of healthcare professionals to communicate clearly, concisely, and empathetically is central to effective clinical practice [2]. Despite advancements in medical technology and treatment protocols, communication failure remains one of the most persistent and preventable causes of medical error [3]. This issue is especially critical in high-pressure settings such as emergency departments, operating rooms, and intensive care units, where breakdowns in communication can lead to life-threatening consequences [4].

Recognizing its vital role in patient care, healthcare institutions have increasingly integrated communication training into both undergraduate medical education and continuing professional development programs. However, traditional instructional formats such as lectures, written case studies, and basic role-play often lack the realism and unpredictability of actual clinical encounters [5]. These approaches may not fully equip learners to manage the emotional, relational, and contextual challenges that define real-world healthcare communication.

To address this gap, educators have turned to experiential learning as a method that immerses learners in structured, real-life scenarios to strengthen interpersonal skills and adaptability. One such approach gaining momentum is adventure-based experiential learning (AEL), which places participants in physically and mentally engaging team challenges aimed at developing communication, leadership, and collaboration under pressure [6]. AEL emphasizes real-time decision-making and dynamic teamwork, mirroring the demands of modern clinical practice [7].

As healthcare continues to shift toward interprofessional, team-based models of care, communication is increasingly recognized not as a static skill but as an adaptive process shaped by context, culture, and situational urgency. Miscommunication among physicians, nurses, allied health professionals, and support staff can result in fragmented care, redundancies, and adverse outcomes [8]. In contrast, effective communication builds trust, ensures accurate information transfer, and enhances team-based decision-making [9]. Training approaches like AEL, which simulate the complexities of clinical collaboration in a controlled yet challenging environment, offer a promising strategy for preparing professionals for real-world communication demands [10].

This study aims to evaluate the impact of a one-day AEL intervention on the communication competence of healthcare professionals working in a tertiary care setting. Specifically, it examines whether experiential learning activities can improve communication adaptability, social awareness, and teamwork. This research seeks to generate practical insights to guide the development of future communication training programs by recording pre- and post-intervention outcomes, and contribute to improved clinical collaboration and patient care.

Significance of communication in healthcare

Communication is more than a soft skill in healthcare settings; it is a core clinical competency that directly impacts patient safety, health outcomes, and system efficiency [1]. Miscommunication remains one of the leading contributors to preventable adverse events, including diagnostic delays, treatment errors, and procedural complications [2]. These breakdowns often arise not from a lack of technical knowledge, but from unclear messaging, poor listening, or hierarchical barriers within clinical teams. In contrast, effective and empathetic communication fosters trust, improves patient satisfaction, and enhances adherence to treatment plans [3].

Healthcare environments are often high-stakes and fast-paced, where timely and effective information exchange is critical. The ability to communicate with composure, empathy, and clarity under pressure is not innate; it must be taught, practiced, and refined over time [4]. Yet, traditional medical education has historically prioritized biomedical content over interpersonal competencies, leading to a gap in how providers are prepared for emotionally charged or complex interactions [5].

Professionals are frequently tasked with leading sensitive conversations around prognosis, end-of-life decisions, cultural preferences, and ethical concerns. These encounters demand more than verbal fluency; they require nonverbal awareness, active listening, and emotional regulation [6]. Without formal training in these areas, clinicians may unintentionally appear rushed, detached, or unapproachable, ultimately compromising the quality of care and patient experience.

Structured communication frameworks such as Situation-Background-Assessment-Recommendation (SBAR), Team Strategies and Tools to Enhance Performance and Patient Safety (TeamSTEPPS), and closed-loop protocols have been widely adopted to improve clarity and reduce errors [7,8]. However, these models primarily focus on the structure and content of communication rather than on the adaptability and emotional nuance needed in unpredictable, emotionally intense situations [9].

Today’s healthcare systems are deeply interdisciplinary, requiring seamless communication between professionals with varied training backgrounds, communication norms, and expectations. When coordination breaks down, it can lead to redundant investigations, conflicting care plans, and unsafe transitions between providers [10,11]. These challenges highlight the urgent need for training programs that go beyond technical accuracy to cultivate interpersonal agility, mutual respect, and collaboration under pressure.

Adventure-Based Experiential Learning (AEL)

AEL is rooted in experiential learning theory, which emphasizes active participation, reflection, and real-time application as the foundations of effective education [1]. Unlike traditional didactic instruction, AEL immerses participants in physically and emotionally engaging scenarios that challenge their communication, decision-making, and team coordination skills [2]. These activities often include outdoor challenges, trust exercises, and team-based problem-solving which are all designed to simulate the urgency, unpredictability, and interdependence of clinical practice.

Kolb’s experiential learning cycle provides the theoretical underpinning for AEL. This model includes four stages: concrete experience, reflective observation, abstract conceptualization, and active experimentation [3]. In AEL, participants face immediate, task-oriented challenges (e.g., navigating a physical course or completing a collaborative puzzle), followed by structured reflection that helps identify patterns in their communication behavior and informs future improvements [4].

One of AEL’s unique advantages lies in its ability to transcend professional roles, encouraging team members to interact as equals. This flattening of hierarchy is especially relevant in healthcare, where rigid structures can sometimes inhibit open feedback or collaborative problem-solving. AEL promotes shared leadership, active listening, and mutual accountability. Notably, these are all critical components of effective clinical teamwork [5].

Participants in AEL settings frequently report increased confidence, adaptability, and awareness of nonverbal communication cues such as body language, tone, and pacing [6,7]. These subtle behaviors are essential to effective patient interaction, especially in emotionally sensitive contexts where verbal communication alone may not suffice.

Within healthcare education, AEL offers a valuable alternative or complement to simulation-based learning, particularly when the training objectives extend beyond technical proficiency to include empathy, emotional intelligence, and resilience [8,9]. Its emphasis on reflection and feedback supports deeper learning, making it a powerful tool for building the humanistic side of clinical practice.

Communication training programs in healthcare

Over the past two decades, communication training has become a central focus of medical education and quality improvement initiatives. While most institutions now include communication competencies in their curricula, the methods used to teach and assess these skills remain inconsistent [1]. Traditional tools such as lectures, case-based discussions, and standardized patient encounters have offered a foundation, but they often fall short in replicating the complexity and unpredictability of real clinical scenarios [2].

Simulation-based training has addressed some of these limitations, offering high-fidelity environments that replicate clinical emergencies and patient interactions. However, even the best-designed simulations can lack the emotional realism and spontaneous collaboration required in actual healthcare delivery [3]. These gaps have led to increasing interest in complementary approaches such as AEL, which focus on the relational and behavioral dimensions of communication in dynamic, often unfamiliar settings [4].

Unlike clinical simulations, AEL removes participants from their routine roles and settings, placing them in challenging team-based tasks that require clear, concise communication, active listening, and collective problem-solving under pressure [5,6]. These scenarios promote critical reflection on personal communication habits, underlying assumptions, and group dynamics, all of which are highly transferable to interdisciplinary clinical practice.

Evidence suggests that AEL can improve interpersonal effectiveness, team cohesion, and interprofessional collaboration which are key elements of safe and coordinated patient care [7]. In diverse care teams, where members bring different perspectives and communication styles, these improvements can reduce miscommunication and improve clinical outcomes.

While frameworks like SBAR and TeamSTEPPS offer valuable structure, they are most effective when complemented by experiential training that helps providers regulate their emotional responses and remain effective in unfamiliar or stressful contexts [8,9]. Unlike structured models like SBAR or TeamSTEPPS, which standardize communication content, AEL focuses on adaptability, emotional regulation, and real-time decision-making. The two approaches are complementary; however, no formal integration was included in this intervention. AEL fills this educational gap by promoting emotional resilience and authentic dialogue even in challenging team situations. 

Communication training must move beyond checklists and structured scripts toward deeper, behaviorally grounded skill-building. Integrating AEL into professional development and academic programs offers a strategic opportunity to cultivate healthcare professionals who are both clinically competent and communicatively agile, emotionally intelligent, and prepared for the realities of modern patient care [10,11].

## Materials and methods

Study design

This study employed a quasi-experimental, pre-test/post-test design with two parallel groups: an experimental group that received the intervention and a control group that underwent standard communication training. The objective was to evaluate the effectiveness of a one-day AEL program in enhancing communication competence among healthcare professionals. The study was conducted over three months at Sree Balaji Medical College & Hospital, a tertiary care hospital in Chennai, India. Data were collected at two time points: before the intervention (pre-test) and one week after the intervention (post-test) to measure changes attributable to the AEL program.

Participants and sample size

A total of 500 healthcare professionals participated in the study, including physicians, nurses, and allied health staff employed at the same institution. Participants were randomly assigned to either the experimental group (n=250, 50%), which received the AEL intervention, or the control group (n=250, 50%), which continued with existing communication training. Participants were randomized using a computer-generated allocation sequence. Group assignments were concealed until the intervention day to reduce allocation bias. Participation was voluntary, and all consenting individuals were equally eligible, minimizing selection bias.

Inclusion criteria required participants to be healthcare professionals aged 23 years or older, with at least one year of clinical experience in a hospital setting, and willing to provide written informed consent. Participants were drawn from diverse departments, including emergency medicine, surgery, internal medicine, and nursing services, to ensure interdisciplinary representation.

Exclusion criteria included prior participation in any AEL program within the past year, refusal to participate, or inability to complete both the pre- and post-intervention assessments.

Baseline demographic characteristics such as age, gender, and professional role were comparable between the experimental and control groups, thereby supporting internal validity and group equivalence for analysis.

Intervention: AEL program

The AEL intervention was designed to enhance communication, leadership, adaptability, and teamwork through structured outdoor activities. Conducted at an off-site training facility, the one-day program included a progression of team-based exercises aimed at simulating communication in high-pressure environments.

The AEL session commenced with a series of structured icebreaker activities aimed at fostering psychological safety and interpersonal familiarity among participants. These were followed by trust-building exercises such as blindfolded guidance and paired balance tasks that emphasized the importance of verbal precision, active listening, and mutual reliance. Mid-day activities included time-bound team navigation tasks and scenario-based challenges that simulated clinical decision-making under pressure. These tasks required participants to negotiate roles, delegate responsibilities, and adapt strategies in real time. In the afternoon, participants undertook low-rope elements including the spider web and balance beams, as well as high-rope components such as suspended bridges and climbing towers. These physically and mentally engaging tasks demanded clear safety communication, rotating leadership, and emotional regulation. The program concluded with a structured facilitator-led debriefing session in which participants reflected on their own and others’ communication styles, identified strengths and weaknesses in teamwork, and formulated actionable takeaways applicable to their clinical settings.

In contrast, the control group received the hospital’s standard communication training, which consisted of asynchronous online modules and instructor-led classroom sessions focused on clinical communication frameworks. 

Measurement tool

Communication competence was assessed using the CAS developed by Duran (1983) [11]. This validated self-report instrument measures six domains of communication adaptability (Table 1)

**Table 1:** Dimensions of the Communicative Adaptability Scale (CAS)

Dimension	Definition
Social experience	Comfort with diverse social interactions.
Social confirmation	Ability to validate and support others during communication.
Social composure	Ability to manage anxiety and maintain confidence during interpersonal exchanges.
Appropriate disclosure	Judgment in deciding what and how much personal information to share.
Articulation	Clarity and fluency in verbal expression.
Wit	Use of humor to facilitate or manage social interactions.

Each item is rated on a 5-point Likert scale, with higher scores indicating greater competence in that domain. Each domain of the CAS consists of five items scored from one to five, yielding a domain score between five and 25. The total CAS score is the sum of all six domain scores, ranging from 30 to 150. For reporting clarity, scores were divided by five to yield average scores (range: one to five) per domain in the tables. 

The CAS is a validated instrument with strong internal consistency (Cronbach’s α = 0.84-0.89) as reported in prior studies. While originally developed in the US, previous studies in multicultural contexts (e.g., Ruiz-Moral et al., 2019 [12]) have demonstrated its cross-cultural reliability. In this study, no linguistic translation was needed as English is the medium of instruction and communication in the institution.

Data collection and statistical analysis

CAS questionnaires were administered to both groups one week before and one week after the intervention. Pre-test CAS scores were collected one week prior to the intervention. Post-test data were collected one week after the intervention to assess short-term impact. No long-term follow-up assessments were conducted in this study. Data were entered and analyzed using IBM SPSS Statistics for Windows, Version 26 (Released 2019; IBM Corp., Armonk, New York, United States). Paired t-tests were used to compare pre- and post-test scores within each group. Analysis of Covariance (ANCOVA) was applied to compare post-test scores between groups while controlling for baseline differences. Cohen’s d was used to measure effect size, with thresholds of 0.2 (small), 0.5 (medium), and 0.8 (large). A significance level of p<0.05 was considered statistically significant.

Ethical considerations

Ethical approval was obtained from the Institutional Human Ethics Committee (IHEC) of Sree Balaji Medical College & Hospital, Chennai, under Ref. No. 284/SBMCH/IHEC/2024/1987. The study adhered to the Indian Council of Medical Research National Ethical Guidelines (2017) [13] for biomedical and health research involving human participants. Informed written consent was obtained from all participants. Participation was voluntary, and individuals were free to withdraw at any stage. Anonymity and confidentiality were strictly maintained throughout data collection, analysis, and reporting.

## Results

Participant demographics

A total of 500 healthcare professionals participated in the study, with 250 (50%) assigned to the experimental group and 250 (50%) to the control group. The demographic characteristics were well balanced between groups, as shown in Table 2, supporting internal validity.

**Table 2:** Participant demographics (n=500)

Characteristic	Experimental (n=250)	Control (n=250)	Total (n=500)
Gender			
Male	110 (44.0%)	115 (46.0%)	225 (45.0%)
Female	140 (56.0%)	135 (54.0%)	275 (55.0%)
Age (years)			
23–30	85 (34.0%)	90 (36.0%)	175 (35.0%)
31–40	100 (40.0%)	95 (38.0%)	195 (39.0%)
41–50	50 (20.0%)	45 (18.0%)	95 (19.0%)
51+	15 (6.0%)	20 (8.0%)	35 (7.0%)
Profession			
Physicians	80 (32.0%)	78 (31.2%)	158 (31.6%)
Nurses	100 (40.0%)	102 (40.8%)	202 (40.4%)
Allied health workers	70 (28.0%)	70 (28.0%)	140 (28.0%)

Gender representation was nearly even, with 225 male (45%) and 275 female (55%) participants.

In terms of age distribution, the largest group comprised participants aged 31-40 years, a total of 195 (39%) individuals, followed by those aged 23-30 years (n=175, 35%), 41-50 years (n = 95, 19%), and over 51 years (n=35, 7%). Regarding professional background, nurses made up the largest subgroup with 202 (40.4%) participants, followed by physicians (n=158, 31.6%) and allied health workers (n=140, 28%).

Baseline equivalence

There were no statistically significant differences in pre-test CAS scores between the experimental and control groups across all six communication dimensions. This confirms baseline equivalence and supports the use of ANCOVA to analyze post-intervention outcomes.

Effect of AEL on communication competence

The experimental group demonstrated statistically significant post-intervention improvements across five of the six CAS dimensions (p<0.05). The control group, which received standard training, showed only marginal or nonsignificant changes. Results are summarized in Table 3.

**Table 3:** Pre-test and post-test CAS scores CAS: Communicative Adaptability Scale

Dimension	Experimental (Pre)	Experimental (Post)	% Change	Control (Pre)	Control (Post)	p-value (Group effect)
Social experience	3.50 ± 0.47	4.05 ± 0.45	+15.7%	3.48 ± 0.48	3.52 ± 0.46	<0.001
Social confirmation	3.65 ± 0.51	4.15 ± 0.49	+13.7%	3.67 ± 0.50	3.70 ± 0.49	<0.001
Social composure	3.40 ± 0.55	3.97 ± 0.50	+16.8%	3.38 ± 0.54	3.40 ± 0.53	<0.001
Appropriate disclosure	3.58 ± 0.48	3.91 ± 0.46	+9.2%	3.56 ± 0.49	3.58 ± 0.47	<0.01
Articulation	3.46 ± 0.50	3.88 ± 0.49	+12.1%	3.44 ± 0.48	3.46 ± 0.47	<0.05
Wit	3.12 ± 0.54	3.28 ± 0.52	+5.1%	3.10 ± 0.55	3.11 ± 0.54	0.12 (NS)
Overall CAS score	3.47 ± 0.50	3.88 ± 0.48	+11.8%	3.45 ± 0.49	3.48 ± 0.48	<0.001

ANCOVA: Adjusted post-test scores and effect sizes

An ANCOVA was conducted to adjust for any pre-test differences and assess the true effect of the intervention. Adjusted mean post-test scores and Cohen’s d effect sizes are reported in Table 4.

**Table 4:** ANCOVA results for post-test CAS scores CAS: Communicative Adaptability Scale; ANCOVA: Analysis of Covariance.

Dimension	Adjusted mean (Exp.)	Adjusted mean (Control)	Mean difference	p-value	Effect size (Cohen’s d)	95% CI for d
Social experience	4.03 ± 0.05	3.52 ± 0.05	0.51	<0.001	0.82 (Large)	(0.71, 0.93)
Social confirmation	4.12 ± 0.05	3.70 ± 0.05	0.42	<0.001	0.78 (Large)	(0.67, 0.89)
Social composure	3.95 ± 0.0	3.40 ± 0.05	0.55	<0.001	0.85 (Large)	(0.73, 0.96)
Appropriate disclosure	3.90 ± 0.06	3.58 ± 0.06	0.32	<0.01	0.60 (Moderate)	(0.48, 0.71)
Articulation	3.86 ± 0.06	3.46 ± 0.06	0.40	<0.05	0.58 (Moderate)	(0.46, 0.70)
Wit	3.26 ± 0.07	3.11 ± 0.06	0.15	0.12 (NS)	0.28 (Small)	(0.15, 0.41)
Overall CAS Score	3.88 ± 0.05	3.48 ± 0.05	0.40	<0.001	0.80 (Large)	(0.69, 0.91)

Adjusted post-test means, mean differences, and associated effect sizes with 95% CIs are presented in Table 4. These intervals further affirm the precision and clinical relevance of the observed improvements.

All 500 participants completed both pre- and post-tests. There were no reported dropouts or protocol deviations during the study period. As a result, no imputation or exclusion methods were required in the data analysis. These results validate the hypothesis that immersive, adventure-based training offers a high-impact model for developing communication competence. The observed gains in both affective and cognitive dimensions of communication support the broader implementation of AEL in healthcare education, particularly for enhancing interprofessional teamwork and patient-centered care. 

## Discussion

This study aimed to evaluate the efficacy of an AEL intervention in improving communication competence among healthcare professionals. Findings indicated that AEL can be a powerful, practical strategy for enhancing interpersonal and teamwork-oriented communication skills in clinical settings. A significant increase was observed in CAS scores across five of the six measured domains in the experimental group (n=250; 50%), compared to minimal gains in the control group (n=250; 50%). The largest effect sizes were seen in social experience (Δ=0.55), confirmation (Δ=0.50), and composure (Δ=0.57) (Cohen’s d=0.58-0.85), highlighting both statistical and real-world relevance. 

These changes suggest increased confidence, improved interpersonal responsiveness, and enhanced adaptability in complex interactions. Minimal gains in the Wit dimension (p=0.12) suggest that humor-related communication may require different training methods or a longer intervention duration. This domain showed minimal improvement post-intervention, likely because humor-based communication is more context-dependent and culturally nuanced. As AEL activities did not explicitly target humor or spontaneity, future studies may consider integrating modules specifically aimed at these skills.

The experimental group’s overall CAS score improved by 11.8%, compared to only 0.9% in the control group. These results affirm that immersive, experiential formats may be more effective than traditional lectures or asynchronous training modules, which dominated the control group’s learning pathway.

The ANCOVA results confirmed a statistically and practically significant impact of the AEL intervention on communication competence. Large effect sizes were observed in social composure, social experience, and social confirmation. These dimensions are vital for effective communication in high-pressure, team-based clinical environments. These improvements suggest that participants became more confident, responsive, and adaptable, all of which are essential for collaborative practice.

Moderate gains in appropriate disclosure and articulation further indicate enhanced self-awareness, message clarity, and the ability to adjust verbal expression based on context. In contrast, the dimension of wit did not show significant change, implying that humor-related communication may require more time or a different pedagogical approach to be effectively cultivated. 

Overall, the findings demonstrate that even a single day of AEL can result in measurable and meaningful improvements in key communication skills among healthcare professionals. The most pronounced benefits were seen in domains related to emotional regulation, interpersonal responsiveness, and real-time collaboration. Such skills are often difficult to teach through traditional methods.

These findings align with Kolb’s foundational theory of experiential learning [14], which outlines learning as a cycle involving concrete experience, reflective observation, abstract conceptualization, and active experimentation. In healthcare training, such models have been shown to foster deeper engagement and behavioral change compared to didactic approaches [14,15].

Past research has consistently highlighted the value of experiential learning in health education. Codier et al. [8] demonstrated how emotional intelligence training enhanced the ability of professionals to manage interpersonal stress. This outcome mirrored the gains observed in social composure in our study. Dahlawi et al. [5] and Alhur et al. [4] further emphasized that flattening hierarchical communication and fostering collaboration are vital for safe, high-functioning clinical teams.

Bloemhoff [10] showed that even a one-day AEL program can significantly improve life effectiveness skills in adult learners. Similarly, Skinner et al. [16] found a 20% improvement in communication through small-group experiential learning, which our data also echo. Fejzic et al. [17] demonstrated that simulated modules increased professionalism and communication capacity in pharmacy study. Mata et al. [18] and Moral et al. [19] added that structured communication training, when grounded in self-efficacy frameworks, results in sustained behavioral improvements.

Mechanisms of impact

The observed gains in disclosure and articulation suggest that AEL’s influence extends beyond external communication into reflective and self-regulated behaviors. This may stem from the use of structured debriefing and peer interaction within the AEL model. These elements are identified by Priest et al. [20] and reaffirmed by Byrne et al. [21] as essential for building empathy and trust in healthcare contexts.

Ruiz-Moral et al. [12] reported that communication-focused training programs significantly shift student attitudes and performance, albeit with potential stressors. These sentiments were echoed in further qualitative work by Van Wart et al. [22], where students reported emotional challenges but acknowledged the value of experiential learning.

Through incorporating high-stakes, collaborative scenarios and feedback-rich environments, the AEL format mirrors recommendations by Englar [23] and Newman et al. [15] for immersive, person-centered learning experiences. De Groot et al. [24] found that students in kinesiology similarly benefited from experiential formats, showing increased clarity in communication and team effectiveness.

Practical implications

Integrating AEL into healthcare training, both at undergraduate and professional levels, offers a promising strategy to address known gaps in team communication, patient safety, and staff resilience. Compared to traditional instructional models [3,6], AEL fosters deeper learning and practical skill application. Knight et al. [25] found that dietitians who completed such programs reported a 50% boost in communication confidence, closely aligned with our 11.8% CAS improvement.

AEL offers a scalable model with moderate resource requirements and potential for cross-departmental integration. Compared to simulation-based methods, it is logistically simpler and less dependent on specialized equipment. The one-day format reduces time away from clinical duties, enhancing feasibility in resource-limited settings.

Chorazy et al. [26] illustrated the value of high-impact experiential learning in public health education, noting increased student engagement and knowledge retention. Liao et al. [27] also demonstrated that digital experiential tools can build person-centered communication, especially when physical interactions are limited.

The evidence is further reinforced by Banerjee et al. [28], who showed that well-structured communication programs can enhance the skills of oncology nurses, and Boissy et al. [29], who linked such training to improved patient satisfaction and physician empathy that are both critical metrics in today’s value-based care models.

Taken together, these findings validate the hypothesis that a one-day AEL program can substantially improve communication competence in diverse healthcare professionals. With meaningful gains across multiple CAS dimensions and corroborating support from prior studies, AEL emerges as a viable, scalable training method. Its inclusion in interprofessional education frameworks could help bridge the persistent gap between technical expertise and relational excellence in healthcare practice.

Limitations

This study has several limitations. First, the intervention was conducted over a single day, which may not fully capture the long-term impact of AEL on communication behaviors and the full depth of communication development. Second, communication competence was assessed using a self-reported tool (CAS), which, despite its validation, may be subject to response bias. Triangulating with observational or simulation-based assessments could strengthen future evaluations. Third, the study was conducted at a single tertiary care institution, limiting the generalizability of findings across different healthcare settings. Additionally, while significant improvements were observed in communication domains, this study did not assess the direct impact of these improvements on clinical outcomes such as patient satisfaction, treatment adherence, or error reduction. As the study was conducted at a single tertiary care center in India with a specific institutional culture, the findings may not generalize to different countries or healthcare systems without adaptation. The absence of a long-term follow-up restricts an insight into skill retention over time. Also, participants may have altered their behavior due to awareness of being observed (Hawthorne effect [30]), potentially inflating post-test scores. While the results are promising, caution should be exercised in interpreting the outcomes due to methodological limitations. Future research should replicate the study across diverse institutions and evaluate the longitudinal impact of AEL on clinical performance and patient outcomes. Future research should address the several gaps through multi-center studies, longitudinal follow-up, and inclusion of objective outcome measures.

## Conclusions

The findings from this study provide strong evidence that adventure-based experiential learning is an effective and impactful approach to enhancing communication competence among healthcare professionals. Participants who underwent the AEL intervention showed statistically and practically significant improvements across key dimensions of communication, particularly in areas critical for clinical collaboration, such as social composure, experience, and confirmation. Compared to traditional training methods, AEL offers a dynamic, immersive learning environment that promotes real-time communication, teamwork, and adaptability under pressure. The increase in CAS scores within the experimental group, contrasted with the minimal change in the control group, underscores the added value of experiential learning in health professions education.

Given the increasing complexity of healthcare delivery and the central role of communication in ensuring patient safety, integrating AEL into professional development and medical training programs holds significant promise. Continued research will be essential to confirm these results across varied settings and to explore how improved communication competencies translate into better clinical outcomes and team performance.

## Appendices

Appendix A: Communicative Adaptability Scale (CAS) questionnaire

Instructions to Participants

Please indicate the extent to which you agree or disagree with each of the following statements as they apply to your everyday communication. Use the scale below:

1 = Strongly Disagree 2 = Disagree 3 = Neutral 4 = Agree 5 = Strongly Agree

Social Experience 

(Comfort engaging in a variety of social situations)

I enjoy meeting new people.

I actively seek opportunities to join new groups or teams.

I find it easy to start conversations with strangers.

I am comfortable adapting to different social environments.

I like participating in group discussions.

Social Confirmation

(Ability to validate and support others in conversation)

I show appreciation when someone shares an idea with me.

I make others feel important during discussions.

I acknowledge others’ contributions when we work together.

I often compliment others during interactions.

I encourage others to express their opinions.

Social Composure

(Confidence and calmness during interpersonal interaction)

I can remain calm during difficult conversations.

I speak clearly even when I feel nervous.

I feel confident talking with people in authority.

I stay composed when others challenge my ideas.

I am able to manage anxiety in high-pressure social situations.

Appropriate Disclosure

(Appropriateness of sharing personal information)

I know when to reveal personal feelings in conversation.

I consider the setting before sharing personal details.

I rarely overshare in professional discussions.

I respect boundaries when discussing sensitive topics.

I adjust my level of openness based on who I’m speaking to.

Articulation

(Clarity and fluency of verbal expression)

I speak in a clear and organized manner.

My ideas are easy to understand.

I rarely lose my train of thought while speaking.

I use appropriate words to express my thoughts.

I communicate my opinions effectively.

Wit

(Use of humor to facilitate communication)

I use humor to ease tension in conversations.

I enjoy using light humor to build rapport.

I know how to make others laugh without offending them.

I use humor appropriately in professional settings.

I can diffuse conflict through well-timed humor.

Scoring Instructions

Each domain includes five items. Scores range from 5 to 25 per domain, with higher scores indicating greater communicative adaptability in that area. Total CAS score ranges from 30 to 150.

Appendix B: Adventure-Based Experiential Learning (AEL) program outline

Program Title

Enhancing communication competence through experiential learning

Duration

One day (eight hours).

Location

Designated outdoor training facility affiliated with the host institution.

Program Objectives

Improve communication adaptability across the six CAS dimensions.

Foster interprofessional collaboration.

Build confidence and emotional composure in high-pressure interactions.

Promote self-awareness through reflection and feedback.

Participant Profile

250 healthcare professionals including physicians, nurses, and allied health workers, randomly assigned into small groups of eight to 10 participants per activity cluster.

Program Schedule

(Table 5)

**Table 5:** Schedule and activity components CAS: Communicative Adaptability Scale

Time	Activity	Purpose
08:30 – 09:00 AM	Welcome and orientation	Program goals, safety briefing, team assignment
09:00 – 09:45 AM	Icebreaker games	Establish rapport, reduce anxiety
09:45 – 11:00 AM	Trust-building tasks	Enhance active listening, mutual reliance
11:00 – 12:30 PM	Problem-solving challenge I	Practice articulation and collaboration
12:30 – 01:30 PM	Lunch break	Informal peer bonding
01:30 – 03:00 PM	Low-ropes course	Improve real-time verbal coordination
03:00 – 04:30 PM	High-ropes challenge (optional)	Build composure, manage communication under stress
04:30 – 05:30 PM	Group reflection & guided debrief	Analyze communication behaviors, give/receive feedback
05:30 – 06:00 PM	Closing & evaluation	Share insights, complete post-test CAS

Facilitation

Conducted by certified AEL trainers with clinical simulation experience. On-site medical safety team and facilitators for each activity. Emphasis on psychological safety, inclusivity, and experiential learning principles.

Evaluation

Pre- and post-intervention assessment using CAS. Verbal feedback collected during debriefing. Written reflections submitted anonymously post-session.
